# Improved classification of breast cancer peptide and protein profiles by combining two serum workup procedures

**DOI:** 10.1007/s00432-012-1273-4

**Published:** 2012-07-05

**Authors:** Berit Velstra, Yuri E. M. van der Burgt, Bart J. Mertens, Wilma E. Mesker, André M. Deelder, Rob A. E. M. Tollenaar

**Affiliations:** 1 Department of Surgery, Leiden University Medical Center (LUMC), Albinusdreef 2, 2333 ZA Leiden, The Netherlands; 2 Biomolecular Mass Spectrometry Unit, Department of Parasitology, Leiden University Medical Center (LUMC), Leiden, The Netherlands; 3 Department of Medical Statistics and Bioinformatics, Leiden University Medical Center (LUMC), Leiden, The Netherlands

**Keywords:** Breast cancer, Early detection, MALDI-TOF, Serum proteomics, Magnetic beads

## Abstract

**Purpose:**

Detection of breast cancer at early stage increases patient’s survival. Mass spectrometry-based protein analysis of serum samples is a promising approach to obtain biomarker profiles for early detection. A combination of commonly applied solid-phase extraction procedures for clean-up may increase the number of detectable peptides and proteins. In this study, we have evaluated whether the classification performance of breast cancer profiles improves by using two serum workup procedures.

**Methods:**

Serum samples from 105 breast cancer patients and 202 healthy volunteers were processed according to a standardized protocol implemented on a high-end liquid-handling robot. Peptide and protein enrichments were carried out using weak-cation exchange (WCX) and reversed-phase (RP) C18 magnetic beads. Profiles were acquired on a matrix-assisted laser desorption/ionization time-of-flight (MALDI-TOF) mass spectrometer. In this way, two different biomarker profiles were obtained for each serum sample, yielding a WCX- and RPC18-dataset.

**Results:**

The profiles were statistically evaluated with double cross-validation. Classification results of WCX- and RPC18-datasets were determined for each set separately and for the combination of both sets. Sensitivity and specificity were 82 and 87 % (WCX) and 73 and 93 % (RPC18) for the individual workup procedures. These values increased up to 84 and 95 %, respectively, upon combining the data.

**Conclusion:**

It was found that MALDI-TOF peptide and protein profiles can be used for classification of breast cancer with high sensitivity and specificity. The classification performance even improved when two workup procedures were applied, since these provide a greater number of features (proteins).

**Electronic supplementary material:**

The online version of this article (doi:10.1007/s00432-012-1273-4) contains supplementary material, which is available to authorized users.

## Introduction

With an increasing lifetime risk, currently estimated as one in eight, breast cancer is a leading cause of cancer-related morbidity and mortality (Veronesi et al. 2005). Nevertheless, the mortality rate has decreased over the last decade (Jemal et al. 2009). One of the reasons for this decrease includes early detection through widespread mammography screening (Etzioni et al. 2003). To this end, in many countries, mammography is used as a population-based screening method in women older than 50 years. Unfortunately, up to 20 % of new breast cancer incidents are not detected by this X-ray method, and for younger women with a genetic predisposition, sensitivity is not more than 40 %. Furthermore, specificity of the method is relatively low since only one out of three lesions is found to be malignant (Astley 2004; Benson et al. 2004; Roder et al. 2008). As a result, mammography screening may lead to overdiagnosis (Brennan et al. 2009). From these drawbacks, it becomes obvious that there is an urgent need for novel molecular markers that can improve both sensitivity and specificity for early detection of breast cancer.

A minimally invasive, sensitive, and more specific alternative to mammography could be the use of protein biomarkers in a peripheral blood (serum) test. Mass spectrometry (MS) has shown to be a powerful technology for detection, quantification, and identification of proteins in various body fluids (Aebersold and Mann 2003; Nilsson et al. 2010). So-called MS-based proteomics has benefitted greatly from both instrumental innovations and technological progress in terms of improved resolution, mass accuracy, robustness, and dynamic range of the mass spectrometer as well as from an interest for application in the clinic (Galvao et al. 2011; Ludwig and Weinstein 2005; Palmblad et al. 2009). In a classical profiling study, the aim is to map as many peptides and/or proteins of an individual’s serum or urine sample in one single mass spectrum. These peptide and protein patterns can change as a result of disease and are thus helpful in both early detection and monitoring the development of the disease. However, body fluids are very complex mixtures of biomolecules and therefore require appropriate sample workup (Callesen et al. 2008). In addition to the complexity, the peptide and protein profiles of serum are usually dominated by highly abundant species (Anderson and Anderson 2002). This limitation on the dynamic range can be partly overcome by using advanced separation techniques that yield a defined subset of the proteome. Moreover, new and improved MS systems have been developed to meet the challenge of complexity inherent to biological samples. A promising improvement is to combine various purification methods, thus enhancing the number of detectable peptides and proteins and thereby increasing the odds to find potential biomarkers.

A suitable strategy for protein and peptide extraction is based on the use of functionalized magnetic beads (MBs). Such solid-phase extraction (SPE) has been widely applied for profiling studies in combination with matrix-assisted laser desorption/ionization (MALDI) time-of-flight (TOF) MS (Alagaratnam et al. 2008; Baumann et al. 2005; de Noo et al. 2005, 2006b; Dekker et al. 2005; Jimenez et al. 2007; Nadarajah et al. 2012). For each serum sample fresh, disposable MB-s are used, thus avoiding carry-over that may occur with other techniques such as liquid chromatography (LC). Moreover, MBs with a different functionality allow protein and peptide enrichment based on different chemical–physical interactions, thereby broadening the range of components covered. Various research groups have been evaluating a proteomic profiling approach (Belluco et al. 2007; de Noo et al. 2006a; Fan et al. 2010; Pietrowska et al. 2009; van Winden et al. 2009; Villanueva et al. 2006). Previously, our group has shown that profiles generated from functionalized magnetic beads fractionated serum could differentiate individuals with breast cancer from healthy individuals (de Noo et al. 2006a). In the current study, two different types of MBs, namely weak-cation exchange (WCX) and reversed-phase (RP)C18, were used to generate potentially complementary profiles. The two types of MBs cover a different range of the serum peptidome and proteome, that is, WCX-MBs bind hydrophilic proteins that are mass analyzed up to 11 kDa, whereas RPC18-MBs generally bind (smaller) hydrophobic peptides that are mass analyzed up to 4 kDa. Aiming for an increased sensitivity and specificity for the detection of breast cancer, we have integrated the analysis of WCX and RPC18 profiles. In this study, it will be shown that the classification performance for breast cancer improves when data from two complementary workup procedures are used.

## Materials and methods

**Fig. 1:**
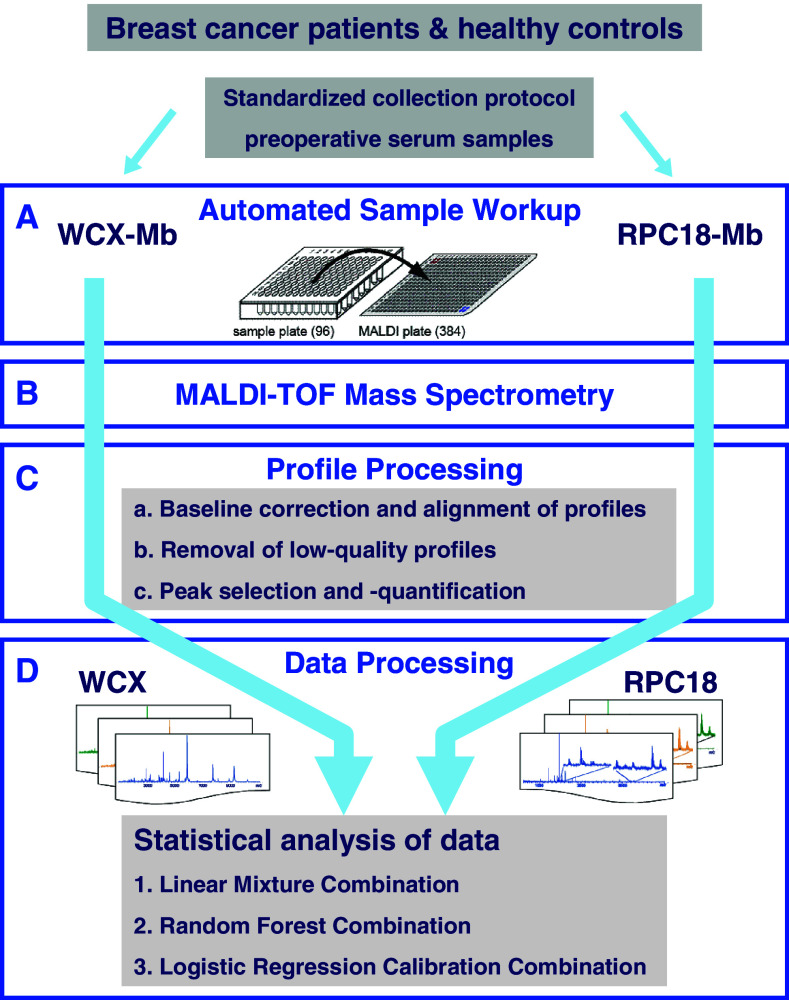
Overview of two sequential processing methods (WCX and RPC18 magnetic beads) for the generation and evaluation of serum peptide profiles. **a** Automated fractionation of samples with both WCX and RPC18 MBs separately. Storage at −80 °C of the MTP’s until measurement. Automated spotting in quadruplicate on MALDI-plate for WCX and RPC18 fractionated samples. **b** Measurements of MALDI-plates in MALDI-TOF. **c** Profile processing: *I* Baseline correction & alignment of four profiles per sample. *II* Removal of spectra without any signal due to spotting failure (max 2.5 %). *III* Selection of interesting peaks and extraction of this data with Xtractor. **d** Data analysis on mean of remaining profiles. Analysis of profiles from the WCX and the RPC18 magnetic bead pipeline separately with LIN and RF. Summarization of the within-bead profiles using double cross-validation predictions. Combination of the predictions of both the WCX and RPC18 data by LIN, RF, and LG analyses

An overview of sample collection, sample and profile processing, and data analysis is depicted in Fig. 1. The sample workup is performed through both a WCX- and an RPC18 MB pipeline.

### Patient characteristics

Serum samples were obtained from 105 female patients with breast cancer (diagnosed by routine pathological analysis) prior to surgery, and from 202 female healthy volunteers (control group) without breast cancer. The median age of the breast cancer patients was 62 years (range, 22–92 years) and of the control group 49 years (range, 18–80 years). Serum samples were obtained in the outpatient clinic of the Leiden University Medical Center (LUMC), The Netherlands, between October 2002 and December 2008. Informed consent was obtained from all subjects, and the study was approved by the Medical Ethical Committee of the LUMC. Female healthy volunteers were accompanying persons.

### Serum samples

Samples were collected and processed according to a standardized protocol: all blood samples were drawn by antecubital venapuncture while the individuals were seated and had not been fasting. For patients, sample collection was performed pre-operative, for healthy individuals at the outpatient clinic. The samples were drawn in an 8.5-cc Serum Separator Vacutainer Tube (BD Diagnostics, Plymouth, UK) and within maximally 4 h centrifuged at room temperature at 1,000 *g* for 10 min (de Noo et al. 2005). Until aliquoting, samples were kept in sterile 500-μl barcode-labeled polypropylene tubes (TrakMate, Matrix TechCorp.) at −80 °C until aliquoting. To this end, samples were thawed on ice in a standardized way and placed in barcode labeled racks in an 8-channel Hamilton STAR® pipetting robot (Hamilton, Bonaduz, Switzerland) for automated aliquoting of 60 μl into daughter tubes. These aliquots were again stored at −80 °C until further sample processing.

### Automated serum workup procedures

Two sequential SPE workup procedures were performed for each serum sample, using only one 60 μl aliquot.

First, 5 μl of serum was used for peptide and protein enrichment with a WCX profiling kit from Bruker Daltonics (Bremen, Germany). This kit contained MBs, as well as binding-, washing- and stabilization-buffers. The manufacturer’s instructions were followed with optimizations that allowed for automation on a 96-channel Hamilton STARplus® pipetting robot, including additional activation and washing steps (Hamilton, Bonaduz, Switzerland). In short, for each sample, a fresh suspension of 10 μl of paramagnetic monodisperse WCX beads was used in a 96-well PCR microtiter plate (MTP) format. WCX-MB binding solution (10 μl) and 5 μl serum sample were added to the beads and carefully mixed using the robot. After 5-min incubation, a magnet was applied for 30 s to allow for optimal settlement of the MBs at the bottom of each well. The supernatant was removed, and the MBs were washed three times with WCX-MB washing buffer. Finally, the peptides and proteins were eluted from the beads using 10 μl custom-made ammoniumhydroxide buffer (NH_4_OH, pH 10). Thus, obtained eluates were transferred to a fresh 96-well eluate plate where WCX-MB stabilization buffer was added (10 μl). Two microliters of the stabilized eluate were transferred to a fresh 384-well mixing plate. Fifteen microliters of α-cyano-4-hydroxycinnamic acid (0.3 g/l in ethanol/acetone 2:1) (MALDI-matrix) was added and mixed carefully by the Hamilton pipetting robot. This mixture was spotted in quadruplicate onto a MALDI AnchorChip™ (600 μm, Bruker Daltonics) target plate using 1 μl for each spot.

Second, 5 μl of serum (from the same aliquot) was used for peptide enrichment with RPC18-functionalized MBs (Jimenez et al. 2007; Nicolardi et al. 2010). For this purpose, 10 μl of RPC18 Dynabeads (Invitrogen) was used for the analysis of 5 μl human serum. The activation, washing and desorption steps of the RPC18 beads were based on the manufacturers protocol and optimized to allow implementation on the 96-channel pipetting robot. For optimal removal of preservatives during the activation step, the RPC18 beads were washed three times with 50 μl of water. For similar reasons, after binding of the peptides to the RPC18 beads, three washing steps with 50 μl of a 0.1 % trifluoroacetic acid (TFA) solution were carried out. Finally, in the peptide desorption step, the remaining peptides and proteins were eluted with 15 μl 50 % acetonitrile (ACN) solution and transferred into a 96-well plate and mixed with stabilization buffer. A portion of these eluates (2 μl) was used for MALDI-spotting. The 96-channel Hamilton pipetting robot was used for mixing of sample eluates with MALDI-matrix, followed by spotting on a MALDI target plate. To this end, 2 μl of the stabilized eluate was transferred into a fresh 384-well MTP and mixed with 10 μl of α-cyano-4-hydroxycinnamic acid (0.3 g/l in ethanol/acetone 2:1). This mixture was spotted in quadruplicate onto a MALDI AnchorChip™ (600 μm) target plate using 1 μl for each of the 384 spots.

### MALDI-TOF–MS peptide and protein profiling

After MALDI spotting, the target plates were immediately placed into a storage chamber (RT, 5 % oxygen, 95 % nitrogen). For MALDI-TOF measurements, each plate was transferred to the mass spectrometer using a robotic system for automated plate loading (Thermo Fisher Scientific Inc., USA). This offers the possibility to carry out all MALDI-TOF measurements within 12 h after spotting using the 96-channel robot. MALDI-TOF mass spectra of the proteins in the WCX eluates were obtained using a positive-ion linear mode acquisition on an Ultraflex III TOF/TOF mass spectrometer (Bruker Daltonics, Bremen, Germany) equipped with a SCOUT ion source and controlled by the Flexanalysis 3.0 software package (Bruker Daltonics). Ions generated by the SmartBeam™ 200 Hz solid-state laser, set at a frequency of 100 Hz, were accelerated to 25 kV and mass analyzed from 960 to 11,024 Da.

MALDI-TOF mass spectra of peptides in the RPC18 eluates were obtained using the same Ultraflex III TOF/TOF mass spectrometer operating in positive reflectron mode in the *m/z*-range of 600–4,000. Sixty laser shots were accumulated for each raster spot, and the sum of 1,200 satisfactory shots, in 60 shot steps, was used for each spectrum (WCX and RPC18). Each profile was obtained after summation of 20 mass spectra. The spectra were externally calibrated using a commercially available peptide mix (Bruker Daltonics). FlexAnalysis Software 3.0 was used for visualization and initial data processing.

### Profile processing

**Fig. 2:**
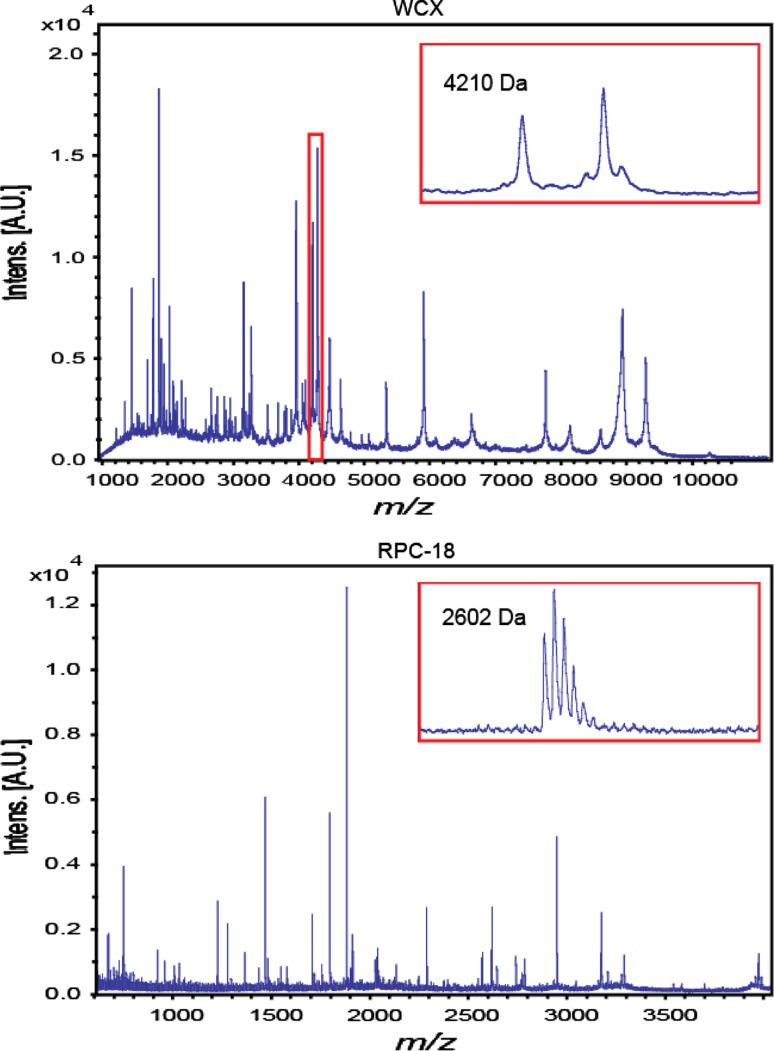
Two examples of the selected peaks for WCX fractionated sample profiles and RPC18 fractionated sample profiles, respectively. On the *x*-axis, *m*/z-values are shown, on the *y*-axis, intensities. Note that WCX-MBs select proteins that are mass analyzed up to 11 kDa, whereas RPC18-MBs generally select smaller peptides up to 4 kDa

In total, 307 serum samples were processed with two types of MBs and MALDI-TOF profiles were obtained in quadruplicate, yielding 1228 WCX- and 1228 RPC18-profiles. For optimal data analysis, all WCX- and RPC18-profiles required baseline correction followed by alignment (see Fig. 1). First, a baseline subtraction of all profiles was performed using the baseline subtraction tool of FlexAnalysis 3.0. Second, to perform the alignment of all 384 RPC18 profiles from one MALDI target plate, at least three peptides at different *m/z*-values were essential for internal calibration. In order to compensate for the possible absence of one or two peptides in a spectrum, the following five peptides were selected based on a manual inspection of a few spectra, namely at *m/z* 1,465.8, *m/z* 1,778.1, *m/z* 1,865.2, *m/z* 2,602.5, and at *m/z* 2,931.5, with a tolerance window of 100 ppm for the *m/z* 1,465.8 peak increasing up to 300 ppm for the highest *m/z*-value (FlexAnalysis 3.0). Similarly, for WCX profiles, the following 7 peaks were visually selected: *m/z* 1,866.1, 3,158.0, 4,643.6, 5,903.7, 6,631.1, 7,765.5, and 9,290.9. In a next step, all low-quality profiles (as a result of failed sample workup or bad MALDI spotting) were excluded from statistical analysis [*n* = 33 for WCX profiles (2.7 %) and *n* = 8 for RPC18-profiles (0.7 %)]. The remaining MALDI-TOF profiles were exported as DAT (.dat) files, all containing *m/z*-values with corresponding intensities. Finally, protein and/or peptide signals in both WCX- and RPC18-profiles were quantified as follows. First, based on visual inspection of the profiles, 48 signals in WCX and 42 signals in RPC18 were selected for further analysis. To this end, a so-called reference file was compiled for both types of profiles including a certain *m/z*-window for each signal or peak. In the WCX profiles, this* m/z*-window reflected the peak width and varied from 5 to 30 Da. In the RPC18-profiles, all peaks were isotopically resolved and of similar shape and width, thus suitable for a fixed window of 0.49 Da. Two examples of selected peaks are shown in Fig. 2. Then, the in-house developed Xtractor tool was used to determine the intensity of each user-defined peak (Selman et al. 2010). This open source tool generates uniform data (peak) arrays regardless of spectral content (ms-utils.org/Xtractor). In the case of RPC18 profiles, peak intensities were determined for each individual isotope of the 42 selected peptide clusters. This detailed information was used for quality control (QC) based on isotopic distribution as reported previously (Nicolardi et al. 2010). For the purpose of this study, the peak intensities of individual isotopes were summed for each isotopic cluster, resulting in a single intensity value for each peptide. The mean of the remaining profiles of the quadruplicate spots for both the WCX and the RPC18 purified samples was used for statistical analysis, as reported previously (Mertens et al. 2011). These processed profiles will be further referred to as the WCX dataset and RPC18 dataset, respectively.

### Data processing and statistics

The WCX- and RPC18-datasets were statistically evaluated as overviewed in Fig. 1. First, a double cross-validatory implementation of linear discriminant analysis (LIN) for the calibration of a diagnostic rule based on a single (mean) spectrum per patient and per magnetic bead was performed, as described previously (de Noo et al. 2006b; Mertens et al. 2006). Next, the predictive performances of both sets (WCX and RPC18) were combined to test whether this improved the performance. For this analysis, the original sets of predictors X^1^ an X^2^ were replaced by the sets of double cross-validated predicted probabilities *p* ^1^ = (*p*_1_^1^, …, *p*_*n*_^1^)^*T*^ and *p* ^2^ = (*p*_1_^2^, …, *p*_*n*_^2^)^*T*^. The predictions *p* ^1^ and *p* ^2^ were combined in a linear mixture combination (MIX). A confirmatory secondary analysis to check the results of the MIX was carried out using the random forest (RF) classification approach as well as using a logistic regression calibration (LG) combination. For each analysis brier score, deviance, sensitivity, specificity, total recognition rate, and area under the curve (AUC) were calculated. This procedure and definitions have been described in detail by Mertens et al. (2011).

## Results

### Classification performance

**Fig. 3:**
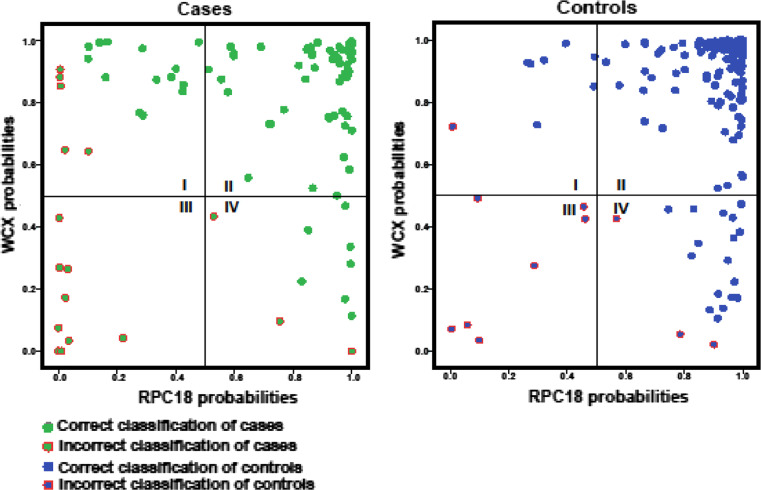
Separate scatter plots for cases and controls versus the double cross-validatory posterior class probabilities calculated from the WCX spectral data (on the *y*-axis) and from the RPC18 spectral data (*x*-axis). For cases, *symbols* are plotted *green* when correctly classified by the LIN and *green* with a *red circle* when otherwise. In the second quadrant, cases are displayed that are correctly classified by both methods. In the third quadrant, cases that are misclassified by both methods are shown. The first and fourth quadrant show discrepancies between the WCX- and RPC18-based assignments. The *green dots* represent the patients that are recovered by the combination. In both of these quadrants, it is clear that there are more correctly assigned cases than incorrectly assigned. For controls, symbols are plotted *blue* when correctly classified and plotted *blue with a red circle* when otherwise

The classification results of WCX and RPC18 datasets were determined for each set separately and for the combination of both sets. In Table 1, the double cross-validatory classification performance measures are shown for WCX and RPC18 datasets independently as well as for the combination of the datasets. As becomes clear from Table 1, all classification performance metrics improved when the combination was compared to the single WCX or RPC18 results. For WCX and RPC18, the total recognition rate using linear discriminant analysis (LIN) was 0.85 and 0.86, respectively, and improved for the integrated datasets in the linear mixture combination (MIX) to 0.91. Likewise, the Brier score improved for the combination WCX and RPC18 [0.084 (MIX) compared to 0.11 WCX (LIN) and 0.11 RPC18 (LIN)]. Furthermore, the deviance improved markedly for the combination. This indicates that not only the total recognition rate improves but also the accuracy of calculation of the class probabilities, which is important from a patient perspective. The improved predictions in the combination also become apparent from two scatter plots of both the WCX- and RPC18- datasets, one including all cases and one all controls (Fig. 3). In these plots, on the left, the incorrectly assigned cases are depicted in the first (upper-left), third (lower-left), and fourth (lower-right) quadrant, with discrepancies between the WCX- and RPC18-based assignments in the first and fourth quadrant. For the cases, there are 29 observations in the first and fourth quadrants of which 21 are classified correctly in the integrated dataset. Subsequently, for the control group, 24 out of 28 observations in these quadrants are recovered by the combination. In the second and third quadrant, there are no discrepancies between both methods for the cases and control groups. In total, there are 234 of such observations, of which 67 in the cases group and 167 in the control group. Only for a few observations, WCX- as well as RPC18-profiles lead to miss-classification (third quadrant), and merging these data does not result in an improved classification. In the case group, these are nine observations and in the control group seven. The classification improvement is due to 45 (21 + 24) out of 57 observations in the first and fourth quadrant shifting to the correct assignment as well as improvement in precision of the calibrated posterior class probabilities. In short, by using two complementary SPE methods, more patients are correctly classified and with higher precision.

**Table 1:** Double cross-validatory classification performance measures from the left to the right for WCX and RPC18 profiles independently and for the combination of WCX and RPC18 profiles (WCX and RPC18)

	WCX 48 peaks	C18 42 peaks	WCX and C18 combination
	LIN	LIN	MIX
Sensitivity	0.82	0.73	0.84
Specificity	0.87	0.93	0.95
Brier	0.11	0.11	0.084
Deviance	242.4	266.8	186.9
Total recognition rate	0.85	0.86	0.91
AUC	0.91	0.89	0.94

### Peak performance

In order to determine the most discriminating peaks, a weighted coefficient of variability of peak expression was calculated for both the 48 peaks selected from WCX profiles as well as the 42 peaks selected from the corresponding RPC18 spectra (For further details, see supplementary file). For WCX, a cut-off point of +2/− 2 of the weighted discriminant coefficient scale was taken and ten peaks remained. Similarly, for RPC18, a selected cut-off point of +3.75/− 3.75 resulted in sixteen discriminating peaks. For WCX and RPC18 separately thus remaining peaks were used to recalibrate the within-bead-based discrimination rule and check whether discriminative performance was maintained after selection. An Independent Student’s *t* test on the case–control data of the ten (WCX) and sixteen (RPC18) peaks was performed for further selection of the most discriminating peaks (*p* values). This test resulted in six significant peaks for discriminating cases from controls for WCX and sixteen for RPC18, respectively. These results, including standard deviations and confidence intervals, are summarized in Table 2. Note that all significant peaks in the RPC18 profiles had a positive *t* value, which indicates that lower expression of these peaks results in a lower chance of being a case.

**Table 2:** Independent samples *t* Test for 10 most discriminating WCX peaks and 16 RPC18 peaks together with the corresponding *m/z*-value in Dalton (Da) as determined from previous identification studies (Tiss et al. 2010)

	Mass	*t* value	SD	*p* value	CI		Identification		Swissprot
WCX									
Peak 4	2,024	−0.8,594	1.0549	0.3908	−0.3588	0.1407	–		
Peak 8	2,770	−6.1616	0.8310	0.000*	−0.8127	−0.4193	–		
Peak 15	3,328	−2.7924	0.9126	0.0056*	−0.5226	−0.0905	–		
Peak 17	3,956	−1.2240	0.9412	0.2219	−0.3614	0.0842	–		
Peak 23	4,480	0.1183	0.8964	0.9059	−0.1995	0.2250	–		
Peak 25	4,963	−3.1870	0.8178	0.0016*	−0.5072	−0.1200	–		
Peak 29	5,248	−3.9337	0.7699	0.0001*	−0.5467	−0.1821	–		
Peak 33	5,920	−6.7727	0.9371	0.000*	−0.9854	−0.5417	–		
Peak 46	8,939	0.3956	1.0182	0.6927	−0.1926	0.2895	–		
Peak 48	10,270	−4.3848	0.9209	0.000*	−0.7038	−0.2678	–		
RPC18									
Peak 3	1,206	4.7685	10.6372	<0.001*	3.5843	8.6208	FGA (5–16)	P02671	
Peak 4	1,211	5.6199	10.9054	<0.001*	4.7917	9.9551	–		
Peak 13	1,449	5.8529	10.7419	<0.001*	5.0209	10.1070	FGA (2–16) H2O	P02671	
Peak 14	1,465	4.6336	10.5497	<0.001*	3.3835	8.3786	FGA (2–16)	P02671	
Peak 23	1,691	5.7363	10.7685	<0.001*	4.8824	9.9811	–		
Peak 25	1,778	6.0120	10.8404	<0.001*	5.2745	10.4073	–		
Peak 26	1,865	6.2572	11.1124	<0.001*	5.7346	10.9961	Complement C3f (1–16)	P01024	
Peak 29	2,021	5.1194	11.0438	<0.001*	4.1875	9.4165	–		
Peak 30	2,271	5.8021	10.8978	<0.001*	5.0273	10.1872	ITIH4	Q14624	
Peak 33	2,602	5.6578	11.0884	<0.001 *	4.9227	10.1728	–		
Peak 36	2,768	4.9575	10.8455	<0.001*	3.9010	9.0362	FGA (576–600)	P02671	
Peak 37	2,931	5.0365	11.1779	<0.001*	4.1269	9.4195	FGA 576–601)	P02671	
Peak 39	3,156	5.5067	11.0767	<0.001*	4.7160	9.9607	ITIH4 (617–644)	Q14624	
Peak 40	3,190	4.9785	10.9766	<0.001*	3.9760	9.1732	–		
Peak 41	3,261	4.8202	11.0915	<0.001*	3.8064	9.0580	FGA (576–604)	P02671	
Peak 42	3,954	5.6858	11.0251	<0.001*	4.9316	10.1518	ITIH4 (645–681)	Q14624	

All *t* values, standard deviations (SD), *p* values, confidence intervals (CI), and identifications with corresponding Swissprot codes are listed in rows

A *p* value < 0.05 was considered significant and marked in this table with *

*FGA* fibrinogen alpha, *ITIH4* inter-alpha-trypsin inhibitor heavy chain H4

## Discussion

Early detection of breast cancer remains a major challenge in medicine. Clinical proteomics has emerged as powerful strategy to develop novel tools for this early diagnosis. In this study, peptide and protein profiles of human serum were obtained aiming at detecting specific patterns present in patients with breast cancer. It was found that peptide and protein profiles can be used to classify breast cancer from healthy control individuals at a sensitivity of 84 % and a specificity of 95 % by using datasets obtained from two complementary SPE methods. Earlier, differentiating protein profiles was reported by our group using C8-functionalized MBs (de Noo et al. 2006a). Although the C8-results were very similar, the current results cannot be qualified as a validation series since the applied MBs were different. Breast cancer serum profiles have been classified with similar success using two other strategies for sample workup, namely with surface-enhance laser desorption/ionization (SELDI) chips and by serum fractionation using a low-molecular-weight cut-off filter (Pietrowska et al. 2009). The importance of controlling the collection of clinical samples, storage conditions, experimental design, spectrometric instruments, and bioinformatics analyses has been stressed in various biomarker discovery studies using MS-based proteomics profiling technology (de Noo et al. 2005; Diamandis 2004; Villanueva et al. 2004). In the current study, we specifically designed our workflow to minimize biases with respect to patient-control handling differences, or variations in sample collection, processing, and storage. A generally recognized pitfall in MS-based profiling studies concerns the large dynamic range in protein concentrations in any body fluid. No single method or instrument can measure *all* proteins in a biological sample, which are typically characterized by a wide range of protein abundances (and often proteins of interest are expressed at a low abundance). Sample fractionation can overcome these issues and reduces the impact of under-sampling and improves reproducibility between analyses (Nilsson et al. 2010). To this end, combining two SPE approaches could result in higher sensitivity for interesting proteins and peptides.

Like in other research fields, at the basis of any well-designed clinical trial lies method development to find the most robust, in this case best reproducible platform for proteomic profiling. In this paper, an improvement at the bioinformatics level is presented by merging data from two MB-strategies. It should be emphasized that the combination of protein profiles, that is, mass spectra, from multiple platforms is not straightforward. The use of support vector machines has resulted in powerful classifications with promising potential for biomarker discovery (Gianazza et al. 2010; Chinello et al. 2010). However, such an approach is not feasible (yet) for combination of MS-based profiles obtained from different platforms. In this work, both low-resolution TOF and high-resolution TOF profiles were used for classification of serum samples and these profiles cannot be simply summed to one single spectrum. Moreover, the combination of data is difficult because this may easily be affected by systematic differences in scaling that causes problems for most standard shrinkage methods, such as dimension reduction-based approaches. In the paper from Mertens et al. (2011), this is further illustrated and clarified. However, data can be merged based on the predictions provided proper cross-validation is applied as shown in this paper. It was previously shown that an approach of linear combination provides a valid model for comparing prediction performance and that furthermore such relatively simple models are preferred with respect to the interpretation of the data (Bovelstad et al. 2007; Hand 2006). Therefore, in this work, the predictions of multiple profiles obtained from the same patient (or control) sample were linearly combined, and it was found that the classification performances improved. An explanation for this improvement could be that the integrated datasets consider more discriminative proteins and/or peptides. However, it may also be that both methods characterize different cleaving products (degradation) from the same protein. In that case, the analysis could be interpreted as if two independent measurements had been performed, and thus, the results improve in a similar way as when taking the mean of two experiments. It is possible that combining the data here presented with other distinct ‘omics’ data will result in an even better discriminating performance. It should be noted that the strategy used in this work for integrating multiple datasets forms a general template for the problem of predictive calibration and that further combinations are currently evaluated.

Generally, in a first step of a peptide or protein profiling study, the diagnostic power of candidate markers is determined. As a second step, identification studies and further investigations into their biological role in disease mechanisms are performed. Note that the identification of peptide or protein signals in a profile is not straightforward. Such efforts require specific separation- or enrichment-strategies as well as a high quality of tandem MS (MS/MS) data for identification of endogenous species, that is, large coverage of fragment ions (Nicolardi et al. 2012). Most reports on serum peptide identifications in profiles are based on SELDI enrichment chips (i.e., Immobilized Metal Affinity Capture (IMAC) or on RPC18 SPE procedures (Nicolardi et al. 2011; Tiss et al. 2010; Villanueva et al. 2006). Considering the sixteen most discriminating RPC18 peaks found in this study (Table 2), these peaks can be matched as fragments of FGA-chain (*m/z*-values at 1,206, 1,449, 1,465, 2,768, 2,931, and 3,261—P02671), fragments of complement C3 (*m/z*-value at 1,865 Da—P01024) and Inter-alpha-trypsin inhibitor (ITIH4) (*m/z*-values at 2,271, 3,156, and 3,954—Q14624) (Tiss et al. 2010). Note that all significant peaks for RPC18 were found with positive *t* values. This is probably due to the fact that peptides under the selected peaks are part of the same protein, as previously described by Villanueva as endoproteolytic cleaving products (Villanueva et al. 2006). It is recognized that inconsistencies in intensity and direction of discrimination and lack of confirmation in other studies make that these proteins as single marker alone have not yet been proven to be reliable for breast cancer identification (Fan et al. 2010). Although a single peptide or protein can be a biomarker molecule in itself, we hypothesize that a full profile is more powerful in terms of specificity. This hypothesis is supported by the fact that in this study all peptides or proteins were found at different discriminating powers as when a larger group of peaks was taken.

## Conclusions

In this study, it was shown that breast cancer MALDI-TOF peptide and protein profiles can be used to classify breast cancer patients from healthy volunteers with good sensitivity and specificity based on the SPE-fractionation using two different magnetic beads. The full automation of the workup procedures ensured a standardized and robust peptide and protein isolation procedure. Combining the data resulting from two complementary workup procedures improved the classification results. The discriminating power or deviance of analyses of both datasets (WCX and RPC18) was very promising compared to conventional mammography results. The integration of datasets from two complementary workup procedures even further improved the classification results. Currently, larger patient sets are analyzed for validation and MS/MS will be used to identify the discriminating proteins and peptides for its use in breast cancer screening programs.

## Electronic supplementary material

Below is the link to the electronic supplementary material.

Supplementary material 1 (DOCX 85 kb)

## Abbreviations

WCX: Weak-cation exchange
MB: Magnetic bead
RP: Reversed-phase
MALDI-TOF: Matrix-assisted laser desorption/ionization-time-of-flight
MS: Mass spectrometry
MS/MS: Tandem mass spectrometry
SELDI: Surface-enhance laser desorption/ionization
IMAC: Immobilized metal affinity capture
SPE: Solid-phase extraction
MTP: Microtiter plate
LIN: Linear discriminant analysis
MIX: Linear mixture combination
RF: Random forest combination
LG: Logistic regression calibration combination
ROC: Receiver operating characteristics
AUC: Area under the curve
