# Supplementary material for: Improved classification of breast cancer peptide and protein profiles by combining two serum workup procedures
Source: J Cancer Res Clin Oncol. 2012 Jul 5;138(12):1983–92. doi: 10.1007/s00432-012-1273-4 (PMC3491194; doi:10.1007/s00432-012-1273-4)
Supplement: Supplementary file 1 — Supplementary material 1 (DOCX 85 kb) [file 432_2012_1273_MOESM1_ESM.docx]

**Supplementary file 1**

Correlation coefficients for both WCX (48) and RPC18 (42) selected peaks. In this figure on the X-axis the number of peaks is displayed, on the Y-axis the discriminating power for a specific peak. A threshold level (red horizontal line) of +2/-2 (WCX) and +3.75/-3.75 (RPC18) resulted in 10 and 16 discriminating peaks, respectively.
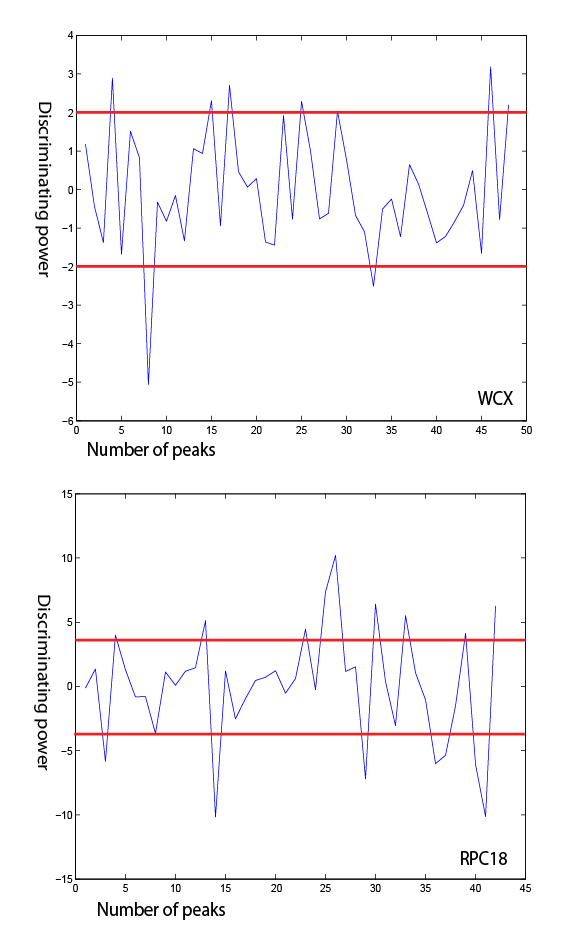


**Supplementary file 2**

Three different scatter plots of the cases: In the first plot on the left a most discriminating peak (peak 33) of WCX data is plotted on the y-axis versus a most discriminating peak (peak 26) of RPC18 data on the x-axis. The scatter plot in the middle represents the linear combination results of the most discriminating peaks of WCX (6 peaks) on the y-axis and RPC18 (16 peaks) on the x-axis. On the right a different configuration from the left plot of figure 3 is shown. The cases are colored dark blue when in the third quadrant and misclassified by both methods. In the first quadrant and thus correctly classified by WCX and incorrectly classified by RPC18 the cases are colored green. Cases correctly classified by RPC18 and incorrectly by WCX are colored light blue (fourth quadrant). In the second quadrant the cases that are correctly classified by both methods are colored red. The same colors for the different quadrants are used in the scatter plot in the middle and on the left. In both the first and the second plot it is clearly that the dark blue dots (and thus the misclassified cases) are again plotted in the third quadrant.


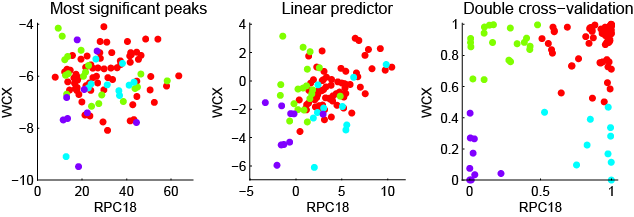


Supplementary Table (extended from Table 1): Classification performance measures from the left to the right for WCX and RPC18 profiles independently and for the combination of WCX and RPC18 profiles (WCX and RPC18), either based on *all* peaks in the profiles (with double-cross validation) or on the most discriminating peaks in the profiles (without double-cross validation).

|  | **WCX**  **48 peaks** | **RPC18**  **42 peaks** | **WCX and RPC18**  **combination**  **(based on 48+42 peaks)** | **WCX 6 most**  **discriminating**  **peaks** | **RPC18 16 most**  **discriminating**  **peaks** | **WCX and RPC18**  **combination**  **(based on 6+16 peaks)** |
| --- | --- | --- | --- | --- | --- | --- |
|  | **LIN** | **LIN** | **MIX** | **LIN** | **LIN** | **MIX** |
| **Sensitivity** | 0.82 | 0.73 | 0.84 | 0.74 | 0.75 | 0.80 |
| **Specificity** | 0.87 | 0.93 | 0.95 | 0.91 | 0.95 | 0.95 |
| **Brier** | 0.11 | 0.11 | 0.084 | 0.11 | 0.095 | 0.084 |
| **Deviance** | 242 | 267 | 187 | 220 | 195 | 179 |
| **Total recognition rate** | 0.85 | 0.86 | 0.91 | 0.85 | 0.88 | 0.90 |
| **AUC** | 0.91 | 0.89 | 0.94 | 0.91 | 0.93 | 0.95 |
